# Splenic artery embolization for refractory ascites after liver transplantation: a single-center experience

**DOI:** 10.1186/s42155-025-00605-3

**Published:** 2025-10-16

**Authors:** Makoto Taninokuchi Tomassoni, Luciana Ingraldi, Paolo Pianta, Alberta Cappelli, Lorenzo Braccischi, Francesco Porta, Antonio De Cinque, Francesco Modestino, Matteo Ravaioli, Matteo Serenari, Federica Mirici Cappa, Maria Cristina Morelli, Matteo Cescon, Cristina Mosconi

**Affiliations:** 1 Department of Radiology, IRCCS Azienda Ospedaliero-Universitaria Di Bologna, Bologna, Italy; 2 Internal Medicine Unit for the Treatment of Severe Organ Failure, IRCCS Azienda Ospedaliero-Universitaria Di Bologna, Bologna, Italy; 3 Department of General Surgery and Transplantation, IRCCS Azienda Ospedaliero-Universitaria Di Bologna, Bologna, Italy; 4 Hepatobiliary Surgery and Transplant Unit, IRCCS Azienda Ospedaliero-Universitaria Di Bologna, Sant’Orsola Hospital, Bologna, Italy

**Keywords:** Embolization, Ascites, Liver, Spleen, Transplant

## Abstract

**Purpose:**

Refractory ascites (RA) is a rare but poorly understood complication following liver transplantation (LT). It is often associated with portal hyperperfusion, potentially driven by splenic hyperafflux. In such cases, splenic artery embolization (SAE) has been proposed as a minimally invasive and cost-effective therapeutic option to reduce splanchnic inflow and alleviate symptoms.

**Materials and methods:**

This retrospective study analyzed patients who underwent LT between August 2010 and September 2023 at IRCCS Azienda Ospedaliera-Universitaria di Bologna and were subsequently diagnosed with refractory ascites. Embolization of the splenic artery was performed using coils or plugs of variable caliber. Laboratory assessments included bilirubin, albumin, alanine transaminase (ALT), aspartate transaminase (AST), alkaline phosphatase (ALP), gamma-glutamyl transferase (GGT), and international normalized ratio (INR). Additionally, Child–Pugh and MELD scores were recorded. The severity and evolution of ascites were monitored through serial ultrasonographic follow-ups.

**Results:**

A total of 12 patients met the inclusion criteria. No severe complications related to the procedure were observed. Among them, 9 patients (75%) experienced complete resolution of ascites and normalization of liver function within 9 months post-procedure. Three patients (25%) died during follow-up due to transplant-related complications unrelated to SAE in the first month after the procedure.

**Conclusion:**

SAE is an effective treatment option for patients with refractory ascites following LT. The procedure resulted in significant improvement in ascites control and liver function in most patients. Good patient selection is essential for a good procedure outcome. Further research with larger patient cohorts and longer follow-up is needed to validate these results.

**Level of evidence:**

Level 3.

## Introduction

In 1996, the International Ascites Club defined “refractory ascites” (RA) as fluid accumulation in the peritoneal cavity that cannot be effectively reduced or whose early recurrence cannot be adequately prevented with medical therapy [1, 2]. Following an orthotopic liver transplant (OLT), this condition has been observed in 3% to 7% of patients [3]. Main causes include hepatic conditions, including alterations in hepatic inflow, outflow obstruction, both acute and chronic rejection, and recurrent hepatitis, as well as extrahepatic conditions such as infection, renal dysfunction, and heart failure. Patients with RA typically experience additional complications such as spontaneous bacterial peritonitis, hepatorenal syndrome, and hepatic hydrothorax, which significantly impact morbidity and mortality, severely affecting patient outcomes [4, 5].

Splenic artery embolization (SAE) has been proposed as a treatment for patients with RA. Previous research has demonstrated that this procedure significantly reduces portal vein (PV) velocity and leads to a decrease in weight, ascites, and the need for diuretics [6–8]. In this article, we present our experience with patients who have undergone SAE for RA.

## Materials and methods

### Study design and population

This was a single-center, retrospective cohort study evaluating the use of SAE as a treatment for RA in patients who underwent OLT. The study included patients treated at IRCCS Azienda Ospedaliero-Universitaria di Bologna between August 2010 and September 2023. RA was defined as persistent or recurrent ascitic fluid accumulation despite optimal medical management, including sodium restriction and diuretic therapy, for at least 3-month post-transplantation.

### Inclusion and exclusion criteria

Patients were eligible for inclusion if they were ≥ 18 years old, had undergone OLT, and developed RA unresponsive to standard medical therapy. Patients with active infection, significant PV thrombosis, or malignancy after OLT were excluded from the study.

### Intervention: diagnosis, laboratory tests, and splenic artery embolization

The pre-procedural evaluation included a triple-phase abdominal CT scan and blood tests, specifically liver function tests—bilirubin, albumin, alanine aminotransferase (ALT), aspartate aminotransferase (AST), alkaline phosphatase (ALP), gamma-glutamyl transferase (GGT), and international normalized ratio (INR). Additionally, creatinine levels were assessed, and Child–Pugh and MELD scores were recorded.

All patients underwent SAE performed by interventional radiologists under fluoroscopic guidance. The procedure was performed via femoral artery access, and embolization was achieved using mechanic embolic agents (e.g., coils and/or plugs).

Based on the clinical context and following multidisciplinary discussion, proximal or superselective partial distal embolization was chosen, with the goal of reducing splenic global arterial inflow while preserving residual splenic function.

Technical success was defined as a significant reduction in splenic perfusion as confirmed by post-procedural angiography.

### Endpoints and outcomes

The primary endpoint was the resolution of RA, defined as a sustained decrease in ascitic fluid accumulation, no longer requiring repeated large-volume paracentesis.

Secondary endpoints included the following:

- *Safety*: Assessed by procedure-related complications and 30-day mortality
- *Liver function*: Evaluated using serial measurements of biochemical markers, including serum bilirubin, ALT, AST, albumin, and INR before and after the procedure

### Follow-up

Patients were followed up at 1-, 3-, 6-, 9-, and 12-month post-procedure. Clinical assessments included the evaluation of ascitic fluid status, liver function tests, and any procedure-related complications.

### Data collection and statistical analysis

Patient demographic and clinical and laboratory data were collected from electronic medical records. Outcomes were analyzed using descriptive statistics. Continuous variables were expressed as mean ± standard deviation (SD) or median (interquartile range, IQR), and categorical variables were expressed as percentages (Tables 1, 2, 3, and 4).

**Table 1:** Patient descriptive analysis before procedure

		*N*	Percentage
**Sex**	F	2	16.7
	M	10	83.3
	Total	12	100.0
**Etiology**	HCV	5	41.7
	HCV, steatosis	1	8.3
	Steatosis	1	8.3
	Steatosis + alcohol	1	8.3
	Alcohol	2	16.7
	HBV + HDV	2	16.7
	Total	12	100.0
**HCC**	No	6	50.0
	Yes	6	50.0
	Total	12	100.0
**Child–Pugh pre**	7	2	16.7
	8	4	33.3
	9	4	33.3
	10	1	8.3
	11	1	8.3
	Total	12	100.0
**MELD pre**	9	1	8.3
	10	2	16.7
	11	2	16.7
	14	1	8.3
	16	1	8.3
	19	1	8.3
	20	1	8.3
	21	1	8.3
	23	1	8.3
	38	1	8.3
	Total	12	100.0
**Caliber of the splenic artery (mm)**	6	1	8.3
	7	5	41.7
	8	4	33.3
	10	2	16.7
	Total	12	100.0
**Splenic ischemia**	No	3	27.3
	Yes	8	72.7
	Total	11	100.0
**Embolization site**	Proximal	5	41.7
	Distal	7	58.3
	Total	12	100.0

**Table 2:** Mean laboratory values before procedure

	*N*	Minimum	Maximum	Mean	Standard deviation
Platelets pre	12	21.00	194.00	66.75	44,866
INR pre	12	1.10	2.22	1.46	0.382
Creatinine pre	12	0.69	4.08	1.51	0.898
Albumin pre	12	2.60	31.80	8.13	10,904
Bil. tot. pre	12	0.40	11.58	3.00	3367
AST pre	12	12.00	4120.00	367.33	1,181,849
ALT pre	12	11.00	3175.00	287.42	909,439
GGT pre	12	15.00	350.00	160.83	113,816
PAL pre	12	50.00	647.00	219.00	153,329

**Table 3:** Lab results after 9 months

	*N*	Minimum	Maximum	Mean	SD
Albumin	6	3.20	4.60	3.89	0.462
Creatinine	7	0.58	1.60	1.14	0.310
INR	6	1.02	2.29	1.30	0.495
Bil. tot	7	0.30	1.70	0.92	0.465
Ast	7	22.00	32.00	27.14	3671
Alt	7	11.00	74.00	33.57	20,509
FAL	7	38.00	380.00	202.71	128,812
NA	7	137.00	146.00	140.57	3259

**Table 4:** Clinical characteristics of the patients after 9 months: classification according to Child–Pugh and MELD scores and assessment of ascites

Child–Pugh 9 months	5	4	66.7
	6	1	16.7
	7	1	16.7
	Total	6	100.0
MELD 9 months	8	2	33.3
	9	1	16.7
	12	2	33.3
	18	1	16.7
	Total	6	100.0
Ascites grade 9 months	Absent	9	75.00
	Missing	3	25.00
	Total	12	100.00

## Results

Twelve patients met the inclusion criteria. Of these, six had hepatitis C virus (HCV)-related liver disease, two had alcohol-related liver disease, two had hepatitis B virus (HBV)-hepatitis D virus (HDV)-related liver disease, and the remaining two had nonalcoholic steatohepatitis (NASH)-related liver disease. Additionally, six patients were diagnosed with hepatocellular carcinoma (HCC) before OLT. All patients developed RA due to portal hypertension following OLT, with portal hypertension confirmed by a portosystemic gradient greater than 10 mmHg, and SAE was proposed as a treatment when other causes of hypertension were excluded.

The mean age at the time of the procedure was 55 years. All patients had splenomegaly (mean craniocaudal length: 173 mm, main volume data pending from CT scans) and normal splenic artery anatomy and origin, with a mean diameter of 7.8 mm. Proximal embolization was performed in five patients, whereas seven patients underwent superselective partial distal embolization. Proximal splenic artery embolization was performed after the origin of the great pancreatic artery, using coils or vascular plugs of variable calibers, oversizing at least 10% for coils and 20% for vascular plugs (from 3 to 14 mm for coils and from 8 to 24 mm for vascular plugs). Total occlusion was confirmed by angiography.

No immediate significant procedure-related side effects were observed after the procedure. Mean AST levels were slightly elevated in the first few days of post-embolization, and imaging revealed signs of splenic infarction in eight patients, mostly in the distal embolization group; however, both findings were clinically insignificant. At the first-month follow-up, all patients showed a significant reduction in ascites, with no ascites present at the 9-month follow-up in nine patients. Liver function normalized within 9-month post-procedure. Three patients, however, died within the months after the procedure due to transplant-related complications unrelated to SAE. At the 1-year follow-up, no recurrence of ascites was observed in the remaining patients.

## Discussion

Although still a debated cause of nonocclusive hepatic flow alteration, splenic artery syndrome (SAS) has been identified as a potential cause of RA in OLT recipients [9]. In 1991, Manner [10] et al. proposed an arterial hypoperfusion of the liver due to a steal phenomenon with blood shunting from the hepatic to the splenic artery as the cause of this condition. However, in 2008, Quintini et al. argued that portal venous hyperperfusion, rather than splenic artery steal, was the underlying etiology of SAS [11]. Despite significant observations made by Quintini’s group, they were unable to establish causality, and the pathophysiology of SAS remains controversial [12]. No study has yet proven causality, and the hemodynamics influencing liver transplant perfusion are complex and multifactorial [13].

A patient with cirrhotic liver has an altered systemic and splanchnic circulation that is not immediately restored after transplantation, considered one of the main causes of this condition. The contribution of splenic blood to the PV flow in patients with cirrhosis can be as high as 60% [14–16]. SAE has been proposed as a minimally invasive safe and effective treatment for RA [17]. Splenic artery occlusion reduces blood flow to the spleen and splenic vein, lowering PV pressure gradients and alleviating portal hypertension. Several case reports [18–21] have demonstrated that splenic artery embolization for posttransplant ascites can reduce PV velocity, decrease diuretic dependence, promote weight loss, and achieve complete resolution of ascites [22].

One of the earliest and largest reports, presented by Presser et al. in 2015, concluded that SAE could become the first-line treatment for RA [7]. Proximal SAE is preferred over distal SAE, as it decreases blood flow while allowing distal revascularization, reducing the risk of complications [23]. In 2022, Fleckenstein et al. suggest that long-term outcomes following SAE for SAS after LT are not influenced by the embolization site. However, proximal embolization may promote earlier normalization of liver function [24].

This study represents a single-center experience with the use of SAE as a treatment for RA following OLT. Our results demonstrate that SAE is a promising intervention for patients with RA due to portal hypertension, with favorable outcomes in terms of both ascites resolution and liver function recovery.

Our study found that SAE led to a significant reduction in ascites in all patients, with nine patients achieving complete resolution of ascites by the 9-month follow-up. This is consistent with previous studies that have shown the effectiveness of SAE in treating portal hypertension-related complications, such as ascites and variceal bleeding. The improvement in ascites was sustained, with no recurrence observed in the remaining patients at the 1-year follow-up. The resolution of ascites in this cohort suggests that SAE can effectively reduce the portal hypertension that drives ascitic fluid accumulation, providing long-term benefits for patients.

The safety profile of SAE in this cohort was favorable, with no immediate procedural related complications and only mild, clinically insignificant post-procedural changes in liver enzymes and imaging findings of splenic infarction in a subset of patients. This is consistent with previous reports, which have suggested that SAE is a relatively safe procedure, with complications typically limited to mild splenic infarction and occasional post-procedural pain. Importantly, no patients experienced significant bleeding, infection, or other serious adverse events, highlighting the low risk of SAE when performed by experienced interventional radiologists. Nevertheless, a high mortality within the first 30 days was seen after the procedure. This might be explained by the fragility of these patients, and two patients died by hepatorenal syndrome and the other due to complications from a strangulated inguinal hernia. These outcomes suggest that renal function, along with the patient’s underlying conditions, should be considered a key factor in determining the suitability of SAE to achieve more favorable post-procedural outcomes.

Despite the promising results, several limitations of this study should be acknowledged. First, the small sample size and retrospective design limit the generalizability of our findings. Additionally, the lack of a control group makes it difficult to determine the relative effectiveness of SAE compared to other treatment options, such as TIPS or repeated paracentesis. Furthermore, the long-term effects of SAE on liver function and survival were not fully evaluated, as the follow-up period was limited to 1 year. Larger, prospective, and multicenter studies are needed to better assess the role of SAE in the management of RA after OLT, as well as its long-term safety and impact on liver function.

## Conclusion

Our study suggests that SAE is an effective treatment option for patients with RA following LT. The procedure resulted in significant improvement in ascites control and liver function in the majority of patients. Further research with larger patient cohorts and longer follow-up is needed to validate these results.

## Data Availability

The datasets used and/or analyzed during the current study are available from the corresponding author on reasonable request.
